# Common molecular mechanism of amyloid pore formation by Alzheimer’s β-amyloid peptide and α-synuclein

**DOI:** 10.1038/srep28781

**Published:** 2016-06-29

**Authors:** Coralie Di Scala, Nouara Yahi, Sonia Boutemeur, Alessandra Flores, Léa Rodriguez, Henri Chahinian, Jacques Fantini

**Affiliations:** 1 Aix-Marseille Université, PPSN-EA4674, Faculté des Sciences, 13013 Marseille, France

## Abstract

Calcium-permeable pores formed by small oligomers of amyloid proteins are the primary pathologic species in Alzheimer’s and Parkinson’s diseases. However, the molecular mechanisms underlying the assembly of these toxic oligomers in the plasma membrane of brain cells remain unclear. Here we have analyzed and compared the pore-forming capability of a large panel of amyloid proteins including wild-type, variant and truncated forms, as well as synthetic peptides derived from specific domains of Aβ1-42 and α-synuclein. We show that amyloid pore formation involves two membrane lipids, ganglioside and cholesterol, that physically interact with amyloid proteins through specific structural motifs. Mutation or deletion of these motifs abolished pore formation. Moreover, α-synuclein (Parkinson) and Aβ peptide (Alzheimer) did no longer form Ca^2+^-permeable pores in presence of drugs that target either cholesterol or ganglioside or both membrane lipids. These results indicate that gangliosides and cholesterol cooperate to favor the formation of amyloid pores through a common molecular mechanism that can be jammed at two different steps, suggesting the possibility of a universal therapeutic approach for neurodegenerative diseases. Finally we present the first successful evaluation of such a new therapeutic approach (coined “membrane therapy”) targeting amyloid pores formed by Aβ1-42 and α-synuclein.

Insoluble aggregates of amyloid proteins have been considered for long as the main culprit in Alzheimer’s disease (AD) and Parkinson’s disease (PD)^1^,^2^,^3^. However, this notion has been dismissed during the last decade and instead of these large aggregates small oligomers of Alzheimer’s β-amyloid peptide (Aβ) are now considered the primary neurotoxic species at work in AD and other neurodegenerative disorders, including Creutzfeldt-Jakob, Huntington, and PD^4^,^5^,^6^,^7^. Indeed, there are several documented cases of old people without any typical neurological symptom yet displaying abundant senile plaques in their brain^8^,^9^. Therapeutic approaches of AD based on the clearing of amyloid plaques have failed^10^, and we are now looking for alternative strategies targeting amyloid oligomers^11^. The systematic finding of Aβ oligomers in the brain of AD patients^12^ together with their correlation with AD symptoms^9^ strongly support this new approach. From a structural point of view, amyloid oligomers are spherical^13^, surface-active^14^, and they are prone to form pore-like assemblies in the plasma membrane of brain cells^15^. Such membrane-embedded structures have been initially described as a class of “annular protofibrils” sharing structural similarities with bacterial cytolysins^16^. These annular protofibrils, formed by both Aβ and α-synuclein (the protein associated with PD), were recognized as a new type of “amyloid” assembly and logically referred to as “amyloid pores”^16^. From a functional point of view, amyloid pores behave as Ca^2+^-selective channels responsible for a dysregulated entry of Ca^2+^ in the cytoplasm of brain cells^17^. In this respect, the discovery of amyloid pores^16^ has given a robust structural background for the so-called “calcium hypothesis” of Alzheimer’s disease, a concept that has been initially proposed in the early 1990’s^18^ and has recently gained renewed interest following the failure of the amyloid plaque model to explain the pathogenesis of AD^7^.

The structure of amyloid pores has been extensively studied by ultrastructural methods^16^ including atomic force microscopy^15^, and by *in silico* approaches^19^. Nevertheless, the molecular mechanisms controlling their formation remain mostly unknown. Recently we have shown that cholesterol is required for the assembly of amyloid pores formed by various Aβ peptides (Aβ1-42, Aβ22-35, and Aβ25-35)^20^,^21^. This finding is consistent with *in vitro* studies indicating that Aβ can form ion channels in planar lipid membranes only in presence of at least 30% cholesterol^22^. Independently, it has been shown that gangliosides could control the toxicity of various types of amyloid oligomers^23^ and fibrillar aggregates^24^,^25^. Among these gangliosides, GM1 and GM3 seem particularly interesting to study since they have been involved in the pathophysiology of AD^23^,^26^ and in α-synuclein membrane interactions^27^,^28^. Finally it has been shown that inhibition of cholesterol and ganglioside synthesis could protect cultured neural cells from Aβ toxicity^29^. Using a combination of molecular modelling and physicochemical approaches, we have determined that Aβ and α-synuclein contain both a ganglioside and a cholesterol-binding domain^30^. This finding raised the intriguing possibility of a dual lipid control of membrane permeabilization by amyloid proteins. In the present study, we have used a panel of molecular, physicochemical and imaging approaches to elucidate the roles of these lipids in the formation of Ca^2+^-permeable amyloid pores. We show that both gangliosides and cholesterol are mechanistically involved in the membrane association and oligomeric assembly of Alzheimer’s and Parkinson’s amyloid proteins into functional amyloid pores. Deciphering this universal mechanism of amyloid pore formation allowed us to design and successfully evaluate a common cellular bi-therapy for both Alzheimer’s and Parkinson’s amyloid pores.

## Results

**Figure 1:**
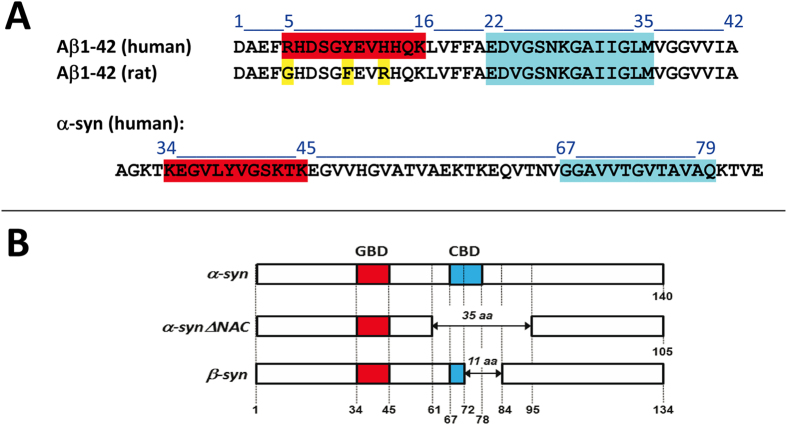
Amino acid sequence and lipid-binding domains in Aβ, α-synuclein and related proteins. (**A**) Amino acid sequence of Aβ1-42 (human and rat) and of the region of human α-synuclein that contains the ganglioside-binding domain (in red) and the cholesterol-binding domain (in blue). Non-conserved amino acid residues in rat Aβ1-42 are in yellow. Note that both the cholesterol-biding domain (fragment 67–79) and the tilted peptide of α-synuclein (67–78) bind cholesterol with high affinity. (**B**) Alignment of α-, β-, and ΔNAC synucleins. Note that all three proteins share the same ganglioside-binding domain (GBD, in red) whereas only α-synuclein displays a complete cholesterol-binding domain (CBD, in blue).

**Figure 2:**
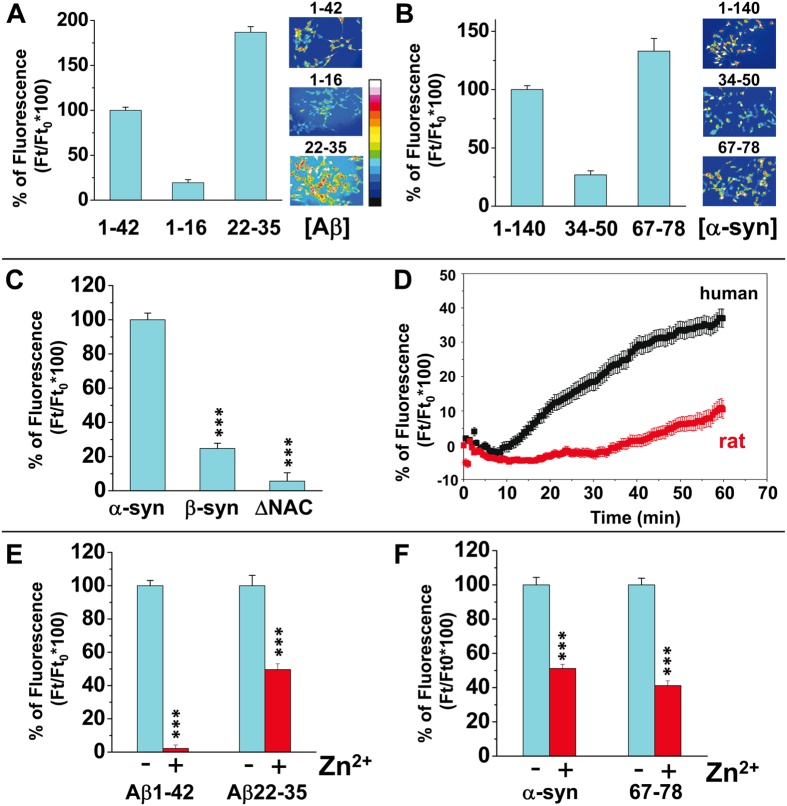
Amyloid pore formation induced by full-length and lipid-binding domains of Aβ1-42 and α-synuclein. SH-SY5Y cells were preloaded with Fluo-4AM, rinsed three times and then incubated with 220 nM of Aβ1-42, Aβ1-16 or Aβ22-35 (**A**), α-synuclein1–140, α-synuclein34–50 or α-synuclein67-78 (**B**), Δ-NACα-synuclein or β-synuclein (**C**) proteins/fragments. The histograms show the mean intensity (±SEM) of the specific Ca^2+^ response measured after 60 (Aβ proteins) or 75 min (synucleins) of incubation (basal Ca^2+^ fluxes did not exceed 5% of the response and were subtracted from all values). The images in panels A and B show pseudocolor representations of cells at the end of the incubation with the protein (scale in panel A). (**D**) Kinetics of intracellular Ca^2+^ concentrations upon treatment with rat (red curve) or human (black curve) Aβ1–42. (**E**,**F**) Effect of the amyloid pore inhibitor Zn^2+^ on Ca^2+^ fluxes induced by Aβ (**E**) or α-synuclein (**F**) proteins. Student’s t-test was used to assess the statistical significance of Ca^2+^-dependent fluorescence between control (full-length Aβ1-42 and α-synuclein) and each experimental condition (e.g. β-synuclein or ±Zn^2+^) α-synuclein and β-synuclein (***p < 10^−4^ with 45 < n < 145).

The formation of functional amyloid pores can be assessed by measuring the entry of Ca^2+^ into amyloid-permissive brain cells^15^,^19^. In this respect, human SH-SY5Y cells have been validated as a reliable model to study amyloid pores generated by nanomolar concentrations of Aβ peptide^20^,^31^. In a typical experiment the cells are preloaded with the fluorescent Ca^2+^-sensitive dye indicator Fluo-4AM and then incubated with the amyloid protein (Supplementary Fig. S1). In the present study, we have analyzed and compared for the first time the pore-forming capability of a large panel of amyloid proteins including full-length, variant and truncated forms, as well as synthetic peptides derived from the ganglioside and cholesterol-binding domains of Aβ1-42 and α-synuclein (Fig. 1). The data obtained upon addition of Aβ1-42 and α-synuclein proteins to SH-SY5Y cells are shown in Fig. 2A,B, respectively. The intracellular concentration of Ca^2+^ induced by each of these proteins is expressed as the percentage of fluorescence increase at the end of the incubation vs. initial conditions. Structure-function relationship studies with variants or synthetic fragments of these proteins were conducted to determine which domains are mandatory for amyloid pore formation. In the case of Aβ, the N-terminal domain Aβ1-16 was totally inactive, whereas the 22–35 fragment (Aβ22-35), previously identified as a cholesterol-binding domain^32^ was fully active (Fig. 2A). Similarly, the cholesterol-binding motif of α-synuclein (fragment 67–78)^30^ retained the ability of the whole protein to generate amyloid pores, whereas the 34–50 fragment was inactive (Fig. 2B). Interestingly, Aβ1-16 and α-syn34–50 display a structurally-conserved ganglioside-binding domain ensuring the initial interaction of amyloid proteins with cell surface gangliosides^33^. This domain displays a typical combination of basic and aromatic residues within a linear segment of 12 amino acid residues, i.e. Aβ5–16 and α-syn34–45 (Fig. 1A). Thus, these data indicate that the cholesterol-binding domains of both amyloid proteins constitute a minimal amyloid-pore forming fragment, whereas the ganglioside-binding domain, by itself, is not able to form a pore. In agreement with the notion that amyloid pore formation is driven by the cholesterol-binding domain, we found that the ΔNAC variant of α-synuclein and β-synuclein, which are either totally or partially deleted in this region^34^ (Fig. 1B), did not form amyloid pores (Fig. 2C). Therefore, a functional cholesterol-binding domain is mandatory to confer an amyloid pore-forming capability. Is it sufficient? The lack of activity of rat Aβ1-42, which differs from the human sequence at only three positions (Fig. 1), all located in the ganglioside-binding domain^35^, suggests that this is not the case (Fig. 2D). In fact, even if the ganglioside-binding domain is apart from the minimal pore-forming domain, its integrity seems to be required for the formation of functional amyloid pores by the full-length protein. Taken together, these data suggest that the assembly of an amyloid pore involves a dual interaction of the protein with gangliosides and cholesterol.

An important control was to assess that the Ca^2+^ response is inherent to the formation of amyloid pores and not to other potential mechanisms such as the stimulation of host Ca^2+^ channels^36^. Therefore, we performed a similar experiment in presence of Zn^2+^ ions which specifically interact with amyloid pores and block their activity^37^. Indeed, Zn^2+^ strongly inhibited or even blocked the Ca^2+^ influx induced by pore-forming Aβ peptides (Fig. 2E) and α-synuclein proteins (Fig. 2F). The difference in Zn^2+^ sensitivity between Aβ1-42 (total inhibition) and Aβ22-35 (partial inhibition) (Fig. 2E) is probably due to the presence of His residues at the pore mouth for the full-length Aβ1-42 protein, but not for the 22–35 fragment which displays amino acid residues (Asp-22 and Glu-23) exhibiting a lower Zn^2+^ affinity than histidine^31^,^38^. Indeed, the lack of His residues at the pore mouth of α-synuclein pores^30^ might also explain why Zn^2+^, used at a concentration of 50 μM (according to Quist *et al*.^15^), induced only a partial inhibition of Ca^2+^ fluxes (Fig. 2F). Despite these discrepancies, a significant inhibitory effect of Zn^2+^ was observed for all Aβ and α-synuclein peptides/proteins, confirming that the Ca^2+^ fluxes induced by these proteins are due to the formation of bona fide amyloid pores.

**Figure 3:**
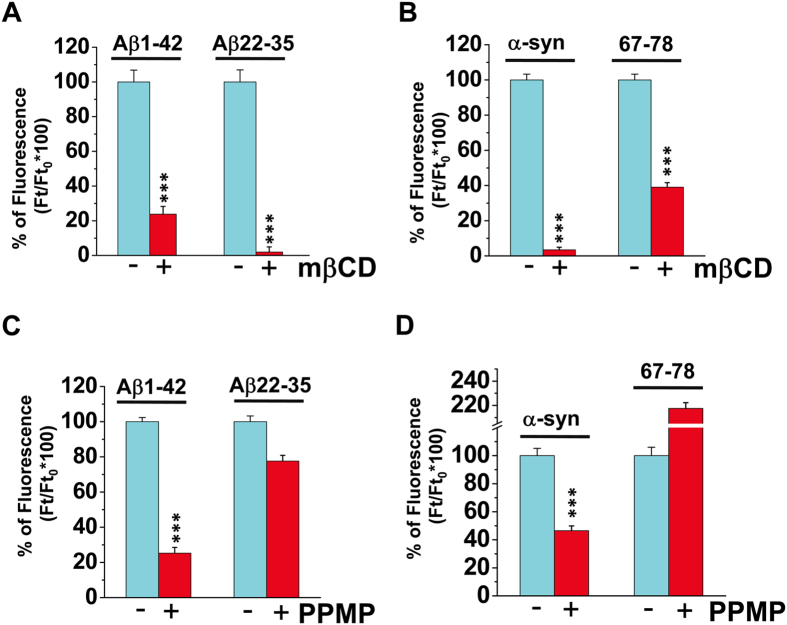
Impact of gangliosides and cholesterol levels for amyloid pore formation. Effect of 1 mM methyl-β-cyclodextrin (mβCD) pretreatment on amyloid pores induced by 220 nM Aβ1-42 or Aβ22-35 (**A**), or 220 nM α-synuclein1-140 or α-synuclein67-78 (**B**). Effect of PPMP pretreatment on amyloid pores induced by Aβ1-42 and Aβ22-35 (**C**) or α-synuclein1-140 or α-synuclein67-78 (**D**) (***p < 10^−9^ with 59 < n < 170). The cells were washed 3 times after mβCD or PPMP treatment, and then again 3 times after the incubation with the Fluo-4AM probe. Under these conditions, mβCD and PPMP were totally removed at the time the amyloid proteins were added to the cells (Supplementary Fig. S2).

Next we analyzed the impact of cholesterol and ganglioside levels on amyloid pore formation. Cholesterol-depleted cells were obtained by mild pretreatment with 1mM methyl-β-cyclodextrin (mβCD) for 24 hours, resulting in a 60% decrease of cholesterol content^20^. In these experiments, the cells were washed three times after the incubation with mβCD, then further washed three times after loading the Fluo-4AM probe. Under these conditions, residual mβCD was below 2 nM after 3 washes and femtomolar after the 6^th^ wash (Supplementary Fig. S2). Thus we could totally exclude the possibility that mβCD could act through a direct interaction with amyloid proteins. The cholesterol-depleting treatment strongly affected the potency of SH-SY5Y cells to sustain amyloid pore formation induced by full-length Aβ (Fig. 3A) or α-synuclein proteins (Fig. 3B). Similarly, the cholesterol-binding fragments of these proteins (Aβ22-35 and α-synuclein67-78) were no longer able to form amyloid pores after the mβCD treatment (Fig. 3A,B). In another set of experiments, decreased expression of gangliosides was achieved by treatment with 1R,2R-(+)-1-phenyl-2-palmitoylamino-3-N-morpholine-1-propanol (PPMP), a metabolic inhibitor of glycosphingolipid synthesis^39^. Under mild conditions (10 μM for 48hr), PPMP induced a global decrease in ganglioside expression as assessed by quantitative analysis of high performance thin layer chromatography plates^40^. Most importantly, PPMP-treated cells had undetectable levels of ganglioside GM1 on their plasma membrane (Supplementary Fig. S3), in full agreement with a recent study^40^. As for mβCD pretreatment, the cells were washed six times between the initial treatment with PPMP and the addition of amyloid proteins. Thus we could rule out a direct interaction between PPMP and amyloid proteins. However, it was difficult to estimate the amount of residual PPMP after washing since the concentrations were below the threshold level of our dosing method. To rule out any possible action of PPMP on amyloid proteins we tested the formation of Aβ1-42 pores in presence of the PPMP concentration used for inhibiting ganglioside synthesis (i.e. 10 μM). Under these conditions, Aβ1-42 fully retained its pore-forming capability (Supplementary Fig. S4). Thus, we could exclude the possibility of a direct binding of PPMP to amyloid proteins so that the effect of PPMP pretreatment could be actually related to lowered ganglioside expression. We observed that PPMP-treated cells were refractory to the formation of amyloid pores induced by full-length Aβ (Fig. 3C) and, although to a less extent, by α-synuclein (Fig. 3D). The weaker effect of PPMP on α-synuclein pores (Fig. 3D) might be due to the involvement of gangliosides such as GM3^33^ that were only partially depleted in PPMP-treated cells. The most remarkable point is that in total contrast with full-length proteins, PPMP did not prevent nor even decrease amyloid pore formation induced by the fragment peptides Aβ22-35 (Fig. 3C) and α-synuclein 67-78 (Fig. 3D), which display the cholesterol-binding domain but lack the ganglioside-binding domain of the amyloid proteins. For α-synuclein67-78, ganglioside depletion resulted in a marked increase of Ca^2+^ fluxes (Fig. 3D), which suggests that this peptide has a higher capability to penetrate the plasma membrane and form amyloid pores when ganglioside synthesis is inhibited. Moreover, one should note that α-synuclein67-78 is more hydrophobic than Aβ22-35 (Fig. 1), so that under conditions where cholesterol is more accessible (PPMP treatment), this peptide may display a higher fusogenic potential. This interpretation is consistent with the established relationship between hydrophobicity and fusogenicity properties of fusion peptides^41^,^42^. Aβ22-35 and α-synuclein67-78 bind to cholesterol but not to gangliosides (Supplementary Table S1). Therefore, the fact that these cholesterol-binding peptides can still form amyloid pores in ganglioside-depleted cells suggests that these short peptides have free access to cholesterol and thus may bypass the ganglioside binding step that is required for the full-length proteins. The involvement of cholesterol in the mechanism of amyloid pore formation by Aβ22-35 and α-synuclein67-78 is also demonstrated by the inhibitory effect of cholesterol depletion (Fig. 3A,B).

**Figure 4:**
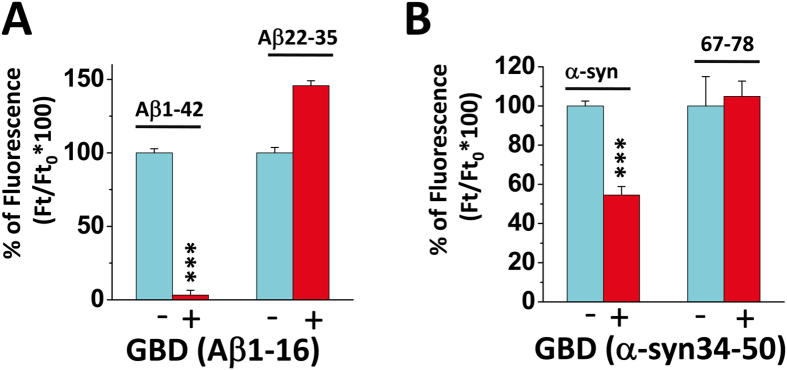
Selective inhibitory activity of synthetic ganglioside-binding domains on amyloid pore formation. **(A)** Aβ1-42 or Aβ22-35 (220 nM) were incubated alone or in competition with the synthetic ganglioside-binding domain (GBD) of Aβ (i.e. Aβ1-16, 220 nM) and intracellular Ca^2+^ concentrations were determined after 60 min of incubation. (**B**) α-synuclein1-140 or α-synuclein67-78 (220 nM) were incubated alone or in competition with the synthetic GBD of α-synuclein (i.e. α-synuclein34–50, 220 nM) and intracellular Ca^2+^ concentrations were determined after 75 min of incubation (***p < ^−9^ with 57 < n < 170).

Next we studied the effects of the isolated ganglioside-binding fragments on amyloid pore formation. We found that the synthetic Aβ1-16 peptide, which encompasses the ganglioside-binding domain of Aβ, efficiently prevented amyloid pore formation by full-length Aβ1-42, but was totally inactive against the cholesterol-binding fragment (Fig. 4A). In these experiments, Aβ1-16 was incubated simultaneously with Aβ1-42. Control experiments were conducted to ensure that the short 1-16 fragment did not interact with the full-length protein. First we studied the interaction of Aβ1-16 and Aβ1-42 with reconstituted GM1 membrane domains (Supplementary Fig. S5). Under our experimental conditions, GM1 was in large excess compared with Aβ. As expected, both Aβ1-16 and Aβ1-42 interacted with the GM1 membrane. When an equimolar mixture of Aβ1-16 and Aβ1-42 was incubated with the membrane, the level of interaction corresponded to the arithmetic sum of the interaction of each individual protein. In presence of a large excess of GM1, these data indicated that Aβ1-16 and Aβ1-42, when mixed together, have fully retained their own avidity for GM1, so that a physical interaction between these proteins in solution is highly unlikely. Another control experiment was conducted to further validate this notion with a distinct approach (Ca^2+^ flux measurements). The cells were preincubated with Aβ1-16 (220 nM), extensively washed, and then incubated in presence of Aβ1-42 (220 nM). In this case, Aβ1-16 was totally removed from the medium before Aβ1-42 is added. Under these conditions, Aβ1-16 still prevented the formation of amyloid pores induced by Aβ1-42 (Supplementary Fig. S6). These data demonstrated that the short ganglioside-binding fragment bound to a cell component (GM1) and not to the amyloid protein. Similarly, the ganglioside-binding fragment of α-synuclein, i.e. α-synuclein34–50, could inhibit amyloid pore formation by full-length α-synuclein, but was without effect on the cholesterol-binding fragment (Fig. 4B). Taken together, these data strongly supported the notion that these cholesterol-binding fragments have direct access to cholesterol and thus bypass the ganglioside binding step that involves the ganglioside-binding domains of each protein. A summary of the cholesterol and ganglioside-binding properties of full-length amyloid proteins and isolated fragments is presented in Supplementary Table S1.

**Figure 5:**
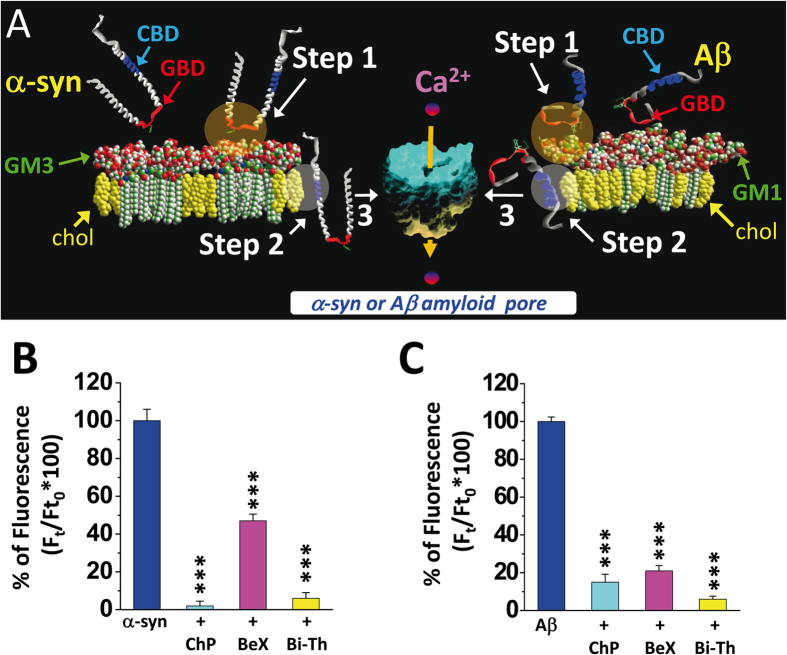
A unique ganglioside/cholesterol bi-therapy formulation against amyloid pores. (**A**) Mechanism of a common ganglioside/cholesterol-dependent pathway of amyloid pore formation by α-synuclein (left pathway) and Aβ1-42 (right pathway). In both cases the mechanism includes an initial interaction (Step 1) with a membrane ganglioside (ideally GM1 for Aβ, GM3 for α-synuclein^33^). This early phase involves the ganglioside-binding domain (GBD) of each protein. Then the protein inserts into the membrane via a cholesterol-dependent process (Step 2) mediated by a cholesterol-binding domain (CBD). Finally the third step (**3**) is the specific oligomerization process of each amyloid protein that leads to the formation of a Ca^2+^-permeable amyloid pore. **(B**,**C).** Cellular therapy for amyloid pores induced by either 220 nM α-synuclein1-140 (**B**) or Aβ1-42 (**C**). In each histogram we compare the effect of a monotherapy with a universal anti-ganglioside peptide (chimeric α-synuclein/Aβ peptide, 220 nM) or with bexarotene (an anti-Alzheimer compound targeting cholesterol, 220 nM), and bi-therapy (mixture of both compounds at 220 nM). In all cases the anti-pore molecules were added simultaneously with Aβ1-42 or α-synuclein1-140 (competition at equimolar concentrations). Legend: ChP, chimeric peptide; BeX, bexarotene; Bi-Th, bi-therapy with both chimeric peptide and bexarotene.

On the basis of these data and on topological considerations of membrane lipids, a molecular model accounting for the formation of amyloid pores is gradually emerging (Fig. 5A). On one hand cholesterol is almost entirely dipped in the apolar phase of the membrane. Thus it is perfectly suited for interacting with the external wall of the pore. This may occur only after the insertion of the cholesterol-binding domain of the amyloid protein in the apolar region of the plasma membrane. On the other hand, the glycone part of gangliosides emerges from the membrane and thus interacts with the extracellular part of the amyloid pore. This extracellular domain contains a series of polar amino acid residues that allows an optimal interaction with host cell gangliosides such as GM1 expressed by neurons or GM3 expressed by astrocytes^7^. Thus, gangliosides may allow the primary attachment of the amyloid protein on the plasma membrane surface. Overall, the formation of an amyloid pore may thus proceed as a two-step mechanism, the first one involving gangliosides and the second one cholesterol. This coordinated process might apply to monomeric proteins^43^ and/or pre-preformed oligomers^15^.

Anyway, given that both gangliosides and cholesterol are required for the formation of amyloid pores by full-length Aβ and α-synuclein, it could be theoretically possible to prevent the formation of these pores by interfering with both ganglioside and cholesterol binding of these proteins. We have tested the effect of a combination therapy with two drugs able to disrupt the two types of lipid-protein interactions that lead to the formation of functional amyloid pores. The first molecule is a short chimeric peptide that combines the ganglioside-binding properties of α-synuclein and Aβ^27^. Briefly, this synthetic chimer is based on the minimal ganglioside-binding domain of α-synuclein, which corresponds to the 34–45 fragment^33^. Since the amino acid residues at positions 42 and 43 are not involved in ganglioside binding, we replaced the wild-type amino acids by a pair of His residues. These His residues efficiently contribute to the ganglioside-recognition properties of Aβ^27^. The resulting chimeric peptide (Supplementary Fig. S7) has an extended ganglioside repertory, and in fact it recognizes all brain gangliosides with high avidity^27^. The second drug of our experimental bi-therapy is bexarotene, a molecule that has been recently tested in animal models of AD and PD with mixed results^44^,^45^,^46^ but is currently under evaluation in patients with Alzheimer’s disease. In fact, our interest for bexarotene was motivated by its chemical resemblance with cholesterol (Supplementary Fig. S7). We recently demonstrated that bexarotene interacts with the cholesterol-binding domain of Aβ and thus blocks amyloid pore formation through competitive inhibition of Aβ-cholesterol binding (Supplementary Fig. S8)^21^. The results of this cellular bi-therapy strategy are summarized in Fig. 5B,C. In the case of α-synuclein, the bi-therapy was superior to bexarotene alone, yet this effect could be attributed to the chimeric peptide which, by itself, totally prevented the formation of amyloid pores (Fig. 5B). However, in the case of Aβ1-42, the bi-therapy appeared superior to each monotherapy as an amyloid pore cure (Fig. 5C). Overall, the combination therapy with both chimeric peptide and bexarotene was the only formulation that proved to be fully active against both Aβ and α-synuclein amyloid pores.

## Discussion

This study was undertaken to determine the respective role of gangliosides and cholesterol in the formation of Ca^2+^-permeable amyloid pores by Alzheimer’s β-amyloid peptides and α-synuclein. To this end we have studied and compared the pore-forming capability of a large panel of full-length, variant and truncated proteins as well as short peptide fragments encompassing the ganglioside and cholesterol-binding domain of these proteins. We have also performed a systematic analysis of the respective effects of ganglioside and cholesterol depleting agents on amyloid pore formation. We have previously shown that PPMP treatment inhibited the formation of amyloid pores by full-length proteins (Aβ1-42 and α-synuclein)^40^. However we did not know if ganglioside depletion could have any effect on short fragments derived from these proteins. Similarly we have reported that Aβ22-35, which display cholesterol-binding properties, could form Ca^2+^-permeable oligomeric pores^20^. Here we show that the minimal cholesterol-binding fragment of α-synuclein (α-synuclein67-78)^30^ also has cholesterol-dependent pore-forming properties. Moreover we have studied the formation of amyloid pores by truncated forms of amyloid proteins (including β-synuclein and ΔNAC α-synuclein which do not display a functional cholesterol-binding domain). All the data presented here strongly suggest that the formation of Ca^2+^-permeable pores in the plasma membrane of brain cells proceeds through a universal mechanism controlled by two types of membrane lipids, gangliosides and cholesterol. Unraveling this complex lipid-driven process allowed to us to develop a new therapeutic approach based on the combination of two inhibitors, one targeting gangliosides and the other one cholesterol. Here we present the first successful evaluation of this new approach (“membrane therapy”) for preventing amyloid pore formation in cultured cells.

Gangliosides, chiefly GM1 and GM3, are recognized by a common structural domain located in the N-terminal part of both Aβ and α-synuclein. Synthetic peptides derived from these segments (Aβ1-16 and α-synuclein34–50) inhibited the formation of amyloid pores induced by the corresponding full-length proteins, i.e. Aβ1-42 and α-synuclein1-140. In contrast, these “ganglioside-binding peptides” did not prevent the formation of amyloid pores by the minimal pore-forming fragments Aβ22-35 and α-synuclein67-78. In this case, the fragments lacked the ganglioside-binding domain and their apolar structure warranted direct access to membrane cholesterol. Correspondingly, the cholesterol-depleting agent methyl-β-cyclodextrin blocked the formation of amyloid pores induced by these fragments. Methyl-β-cyclodextrin also prevented amyloid pore formation induced by full-length Aβ and α-synuclein proteins, indicating that cholesterol is required for pore assembly. In line with these data, mutant Aβ22-35 fragments that did no longer bind cholesterol could not form amyloid pores^20^. Similarly, ΔNAC α-synuclein and β-synuclein, which display deletions of the cholesterol-binding motif, do not form Ca^2+^-permeable pores (Fig. 2C).

The ganglioside-binding domain plays a distinctive role in amyloid pore formation. Sequence variations in the ganglioside-binding region of Aβ affect binding to several gangliosides, including GM1^27,33^. It is the case for rat Aβ, but also for a series of short synthetic mutant peptides derived from Aβ1-16 and α-synuclein34–50^33^. In all cases, the avidity of amyloid proteins for gangliosides involves an electrostatic interaction of basic residues with the negative charge of the sialic acid^33^. Disrupting this interaction with cholera toxin, a classical GM1-binding protein, decreased Aβ oligomer-mediated impairments of long term potentiation (LTP) in mouse hippocampal slices^23^. Similarly, the conservation of the ganglioside-binding domain in β-synuclein (Fig. 1) could explain its inhibitory effect on the formation of amyloid pores driven by α-synuclein^47^. Taken together, these data are in line with the findings of the present study which demonstrate the inhibitory effect of synthetic ganglioside-binding domains (Aβ1-16 and α-synuclein34–50) on amyloid pore formation. The extracellular location of the glycone part of gangliosides strongly suggests that the amyloid proteins bind first to a ganglioside which allows its initial attachment on the plasma membrane surface. Then the protein (either still monomeric or already in an oligomeric assembly) has to insert into the membrane, allowing functional interactions with cholesterol and finalization of the pore assembly process^31^. According to this model, there are two main steps at which the mechanism can be blocked: the initial ganglioside-binding step and the subsequent cholesterol-dependent process (Fig. 5A). This stepwise mechanism is reminiscent of the mechanism of virus fusion, which requires a binding step followed by a post-binding membrane insertion process^30^,^48^,^49^. The analogy between virus fusion and amyloid pores is both structural and functional. Indeed, viral fusion peptides have, like amyloid proteins, both self-aggregating and pore-forming capacities^50^,^51^. From a functional point of view, enveloped viruses such as HIV-1 have developed a complex fusion process that involves the insertion of a fusion peptide in the plasma membrane of the host cell^52^. This penetration is facilitated by the tilted topology of the fusion peptide, a characteristic geometric feature that induces the destabilization of the lipid bilayer during the course of the membrane insertion process^53^. As a matter of fact, this “obliquity-fusogenicity” relationship is a hallmark of viral fusion peptides^54^. Moreover, the tilted geometry of fusion peptides is fully compatible with a physical interaction with cholesterol^31^, an important membrane cofactor of the fusion reaction^55^. In this respect, an initial interaction with plasma membrane glycosphingolipids ensures that the virus envelope fuses with a local domain of the plasma membrane that contains sufficient amounts of cholesterol in the outer leaflet^7^. Since cholesterol and sphingolipids form condensed complexes^56^, binding to a sphingolipid-enriched domain is a warranty that cholesterol is indeed present underneath the sugar head groups of glycosphingolipids^7^. Several studies have shown that HIV-1 fusion is both sphingolipid^57^ and cholesterol-dependent^58^, consistent with the involvement of lipid rafts in HIV-1 infection^59^. From a structural point of view, the dual glycosphingolipid/cholesterol interaction is mediated by two distinct binding domains, one for the glycosphingolipid and the other for cholesterol. This typical structural feature is shared by both virus envelope and amyloid proteins^7^. In particular, the tilted geometry of virus fusion peptides is also a characteristic of pore-forming amyloid proteins, including Aβ1-42^31^,^60^ and α-synuclein^30^,^61^. In the case of amyloid proteins, cholesterol has been shown to induce α-helix formation and to inhibit β-fibrillation^62^. Moreover, *in silico* studies have suggested that the tilted geometry of the membrane-embedded part of amyloid monomers facilitates the oligomerization process through a cholesterol-driven mechanism^20^. Thus, although pore channels based on the oligomerization of amyloid proteins in β-strand configuration have also been demonstrated^15^,^17^,^63^, it is likely that the amyloid pores formed under the control of membrane cholesterol are preferentially α-helical^31^. Future structural studies will help to clarify this point.

In the last part of this study, we have evaluated the potential beneficial effects of a ganglioside/cholesterol-based bi-therapy for preventing amyloid pore formation. The results of this study confirmed that i) the mechanism of amyloid pore formation can be jammed by either ganglioside- or cholesterol-binding agents, and that ii) the combination of both drugs in an experimental bi-therapy provides a unique formulation able to eliminate amyloid pores induced by both Alzheimer’s and Parkinson’s oligomers. In particular, these drugs have additive inhibitory effects and their combination does not induce any adverse or antagonistic activity in cultured cells. The design of this original therapeutic strategy is based on the deciphering of a universal molecular mechanism of amyloid pore formation. As previously discussed, the fact that several distinct amyloid proteins could generate a common oligomer structure^63^,^64^ suggests a common pathogenesis for various neurodegenerative diseases^65^. Our data show that a unique drug formulation, rationally designed for jamming the process of amyloid pore formation in plasma membranes, might be active against distinct neural disorders, including AD and PD. In face of the difficulty to eradicate neurotoxic oligomers^10^, this “membrane therapy” against amyloid proteins considered as “infectious proteins”^7^,^11^ could represent an alternative therapeutic strategy for these diseases.

## Materials and Methods

### Products

SH-SY5Y cells were obtained from the American Type Culture Collection (ATCC). DMEM/F12, HBSS, glutamine and penicillin/streptomycin were from Gibco. Fluo-4AM was purchased from Invitrogen. Full-length amyloid proteins (α-synuclein and Aβ1-42) were from rPeptide. All synthetic peptides were purchased from Schafer (Denmark). The purity of all peptides and proteins is >95% as assessed by high pressure liquid chromatography (HPLC). Aβ1-42, Aβ-22–35 and α-synuclein67-78 were dissolved in 1% NH_4_OH at a concentration of 1 mM and frozen at −20 °C in working aliquots. All other proteins were dissolved in water and stored at −20 °C. The chimeric peptide used in this study^27^ has been patented (PCT/EP2015/054968).

### Cell culture

Cells were grown in Dubelcco’s Modified Eagle Medium: Nutrient Mixture F12 (DMEM/F12) with 10% fetal calf serum, glutamine (2 mM) and penicillin (50 U/mL)/streptomycin (50 μg/mL) and maintained at 37 °C with 5% CO_2_. Cells were passaged twice a week and not used beyond passage 25. When indicated the cells were treated with mβCD (1 mM, 24hr) or PPMP (10 μM, 48 hr) and washed three times with HBSS before further incubation. Under these conditions, the concentration of residual mβCD after the third wash was <2 nM (Supplementary Fig. S2).

### Calcium flux measurements

Intracellular Ca^2+^ levels were measured with the Ca^2+^ sensitive dye Fluo-4AM (5 μM) as described previously^20^. In all cases the cells were washed three times in HBSS after 30 minutes of incubation with the fluorescent probe. A typical calibration experiment is shown in Supplementary Fig. S1. For comparative studies (e.g. Zn^2+^, mβCD, or PPMP treatment), the value obtained with cells treated with the amyloid protein alone was considered as 100% (cyan bars in Figs 2, 3, 4, blue bars in Fig. 5). All experiments were performed at 30 °C during 1 h.

### Statistical analysis

The statistical significance of the data was evaluated with the Student-test or the Kruskal-Wallis test (non-parametric test).

## Additional Information

**How to cite this article**: Di Scala, C. *et al*. Common molecular mechanism of amyloid pore formation by Alzheimer’s β-amyloid peptide and α-synuclein. *Sci. Rep.* **6**, 28781; doi: 10.1038/srep28781 (2016).

## Supplementary Material

Supplementary Information
