# Supplementary material for: Common molecular mechanism of amyloid pore formation by Alzheimer’s β-amyloid peptide and α-synuclein
Source: Sci Rep. 2016 Jun 29;6:28781. doi: 10.1038/srep28781 (PMC4926208; doi:10.1038/srep28781)
Supplement: Supplementary Information [file srep28781-s1.pdf]

Supplementary information

Common molecular mechanism of amyloid pore formation by Alzheimer's β-amyloid peptide and α-synuclein

by Coralie Di Scala, Nouara Yahi, Sonia Boutemeur, Alessandra Flores, Léa Rodriguez, Henri Chahinian and Jacques Fantini

***Fig. S1*: Calcium flux studies.**

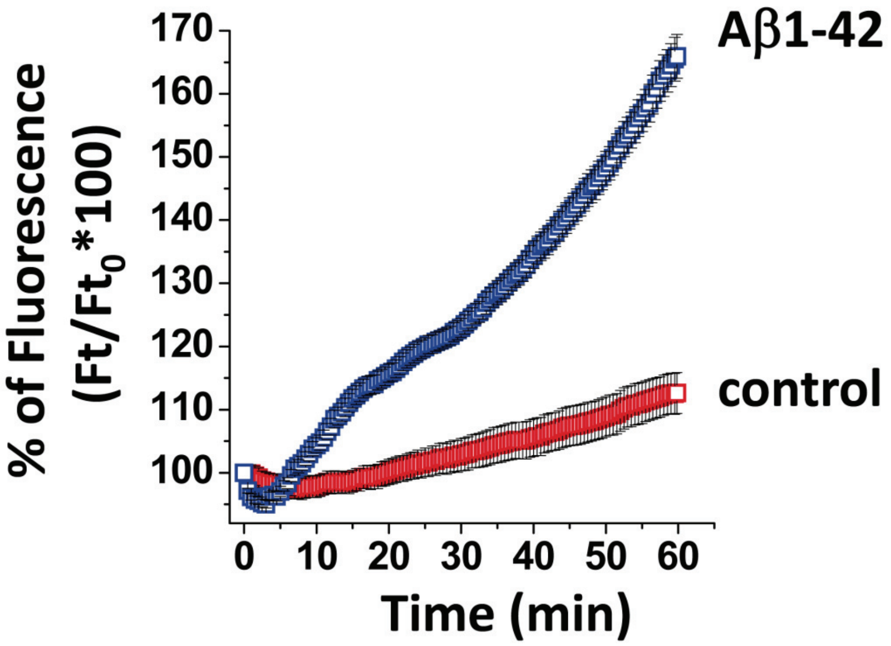

Intracellular Ca^{2+} levels were analyzed in SH-SY5Y cells preloaded with the Ca^{2+} dye indicator Fluo-4AM. In a typical experiment, the amyloid protein (Aβ1-42 in this case) was added at a concentration of 220 nM (blue curve). A control experiment with vehicle alone (either H_20 or 1% NH_4OH) was conducted in parallel (red curve). Signals were expressed as fluorescence after treatment (F_t) divided by the fluorescence before treatment (F_0) and multiplied by 100. The results were then averaged and the fluorescence of control is subtracted of each value. Data are expressed as mean ± S.E.M. (n = 100).

<u>Figure S2</u>: Determination of residual methyl-β-cyclodextrin after two consecutive washes of cell cultures.

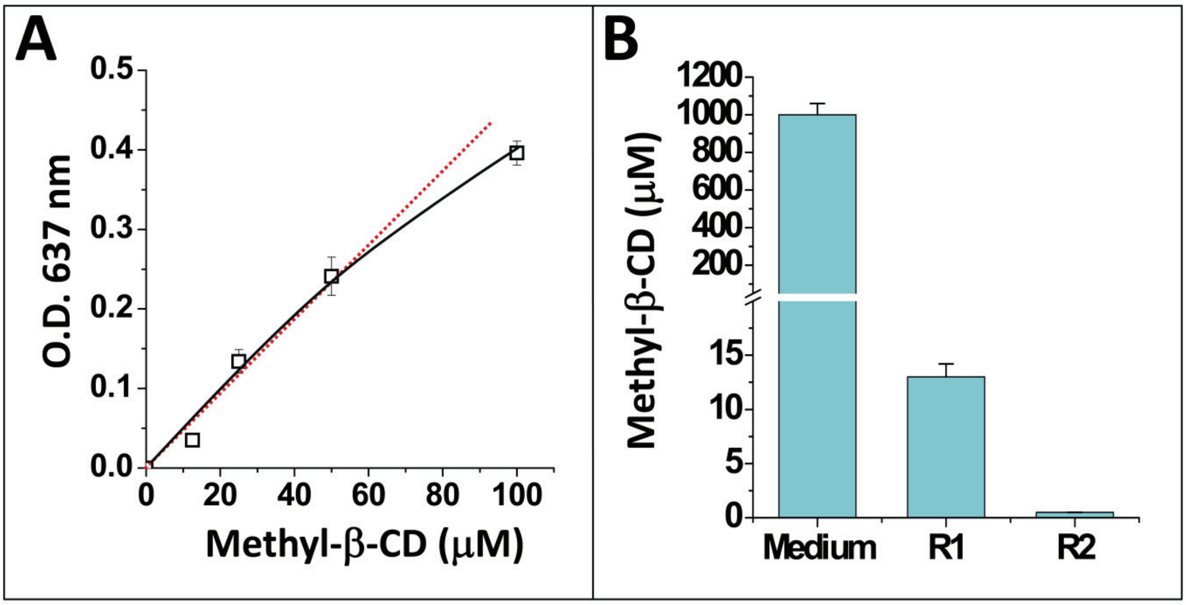

A. Calibration curve of methyl-β-cyclodextrin concentration as determined by the Coomassie Brilliant Blue G-250 method (*Han Liu: A simple, rapid and sensitive method for the quantification of methyl-β-cyclodextrin, available at http://www.paper.edu.cn*). The addition of methyl-β-cyclodextrin to a Coomassie blue solution (100 μM) induces a dose-dependent increase of the optical density (O.D.) at 637 nm. The relationship between absorbance and methyl-β-cyclodextrin concentration is linear over the 0-50 μM range (dotted red line). **B.** Determinations of methyl-β-cyclodextrin concentration in the incubation medium (1 mM) and in the washing medium after the 1st (R1) and 2nd (R2) washes. After the 1st rinse the concentration of residual methyl-β-cyclodextrin is decreased by 83 times (12 vs 1000 μM) to reach undetectable levels after the 2nd rinse. From these data, it can be deduced that after the last (6th) wash preceding the addition of the amyloid proteins, residual methyl-β-cyclodextrin in the incubation medium is as low as 3.10^{-15} M. In fact, only three washes are needed to reach a concentration of 1.74 nM, a value that is far below the concentration of amyloid proteins used in the amyloid pore-forming assay (220 nM). Data are expressed as mean ± S.E.M. (n = 2).

***<u>Figure S3</u>*: Effect of PPMP on cell surface expression of ganglioside GM1.**

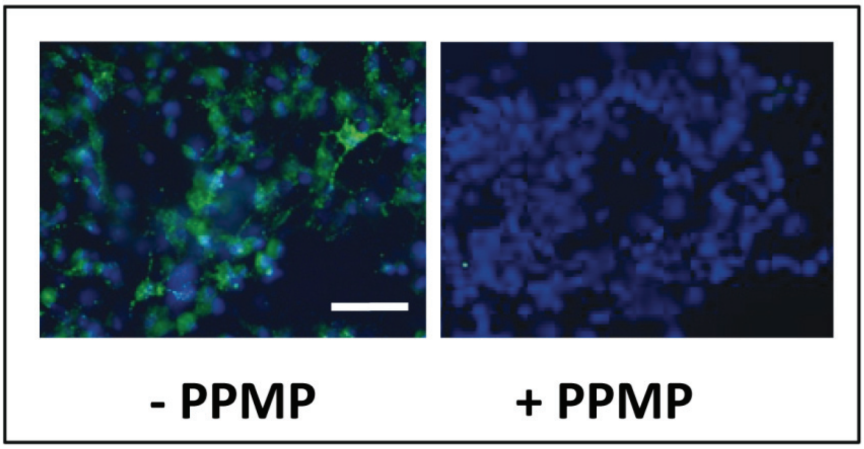

Cell surface detection of GM1 ganglioside was assessed by immunofluorescence staining of control or PPMP-treated SH-SY5Y cells (scale bar = 100 μm). These data showed that PPMP treatment (10 μM, 48hr) induced a dramatic decrease of the cell surface expression of GM1. The anti-ganglioside GM1 antibody was revealed with goat anti-rabbit Alexa Fluor 488.

***<u>Figure S4</u>*: PPMP is not a competitive inhibitor of amyloid pore formation.**

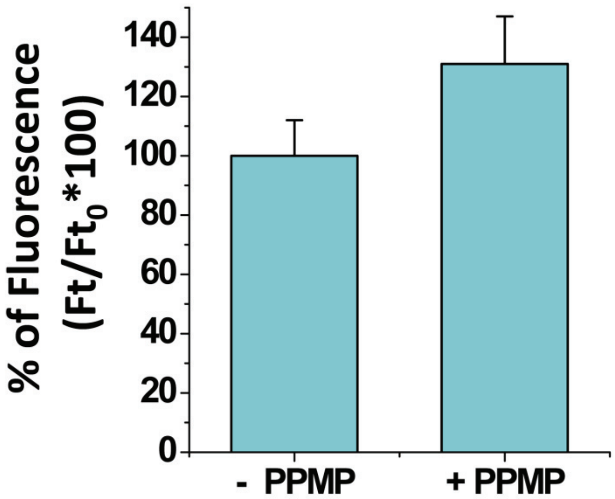

The data show the levels of Ca^{2+} entry induced by Aβ1-42 (220 nM) in SH-SY5Y cells in absence (- PPMP) or presence (+ PPMP) of 10 μM PPMP added simultaneously with the amyloid protein. The intracellular Ca^{2+} concentration was determined after 60 minutes of incubation. Data are expressed as mean ± S.E.M. (n = 40 and 89 respectively for – PPMP and + PPMP conditions).

<u>Figure S5</u>: Additive binding of Aβ1-42 and of the GBD-derived peptide Aβ1-16 on model GM1 containing raft-like domains.

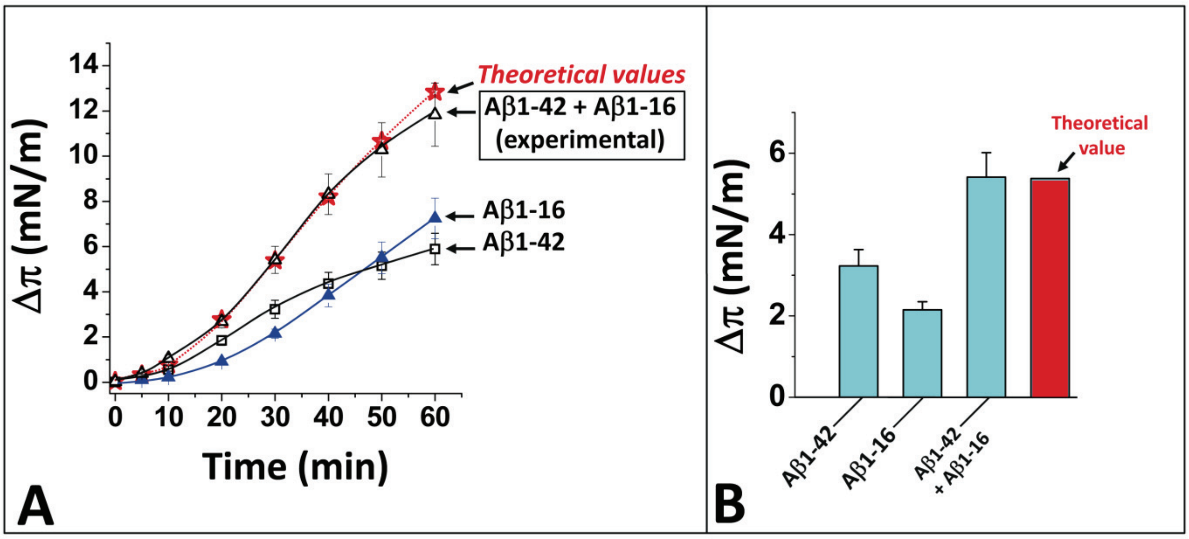

A ternary monolayer consisting of GM1/cholesterol/POPC (1/1/1, mol/mol/mol) was prepared at the air-water interface (initial surface pressure of 20 mN.m^{-1}) as described in Fantini and Yahi (2014)[1]. This reconstituted membrane forms a meniscus with a fixed area. After solvent evaporation, Aβ1-42, Aβ1-16 or an equimolar mixture of both (80 nmol each) was added in the aqueous subphase underneath the monolayer. The interaction with the GM1 raft-like membrane was followed kinetically (**panel A**) or after 30 minutes of incubation (**panel B**) by measuring the surface pressure increase induced by the proteins ($\Delta\pi$). In both panels the arithmetic sum of the surface pressure increase induced by each individual protein is shown in red (theoretical value). Note that in every case the surface pressure increase induced by the Aβ1-42/Aβ1-16 mixture matches the theoretical values calculated by the addition of the $\Delta\pi$ induced by each single protein. These data show that Aβ1-42 and the GBD peptide do not interact together in solution since both bind to GM1, which, in this experiment, is present in large excess in the monolayer compared to the protein concentration. On the surface of neural cells, the amounts of GM1 are much lower so that Aβ1-42 and the GBD peptide would compete for GM1 binding. Data are expressed as mean ± S.E.M. (n = 3). GBD, ganglioside-binding domain; POPC, palmytoyl-oleoyl-phosphatidylcholine.

<u>Figure S6</u>: Preincubation of the cells with Aβ1-16 followed by extensive washings prevents the formation of amyloid pores induced by full-length Aβ1-42.

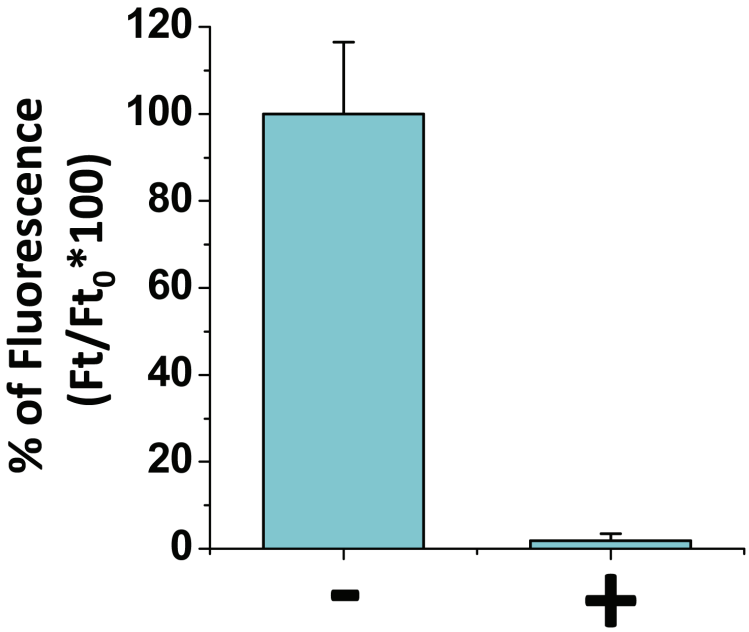

SH-SY5Y cells were preloaded with the Ca^{2+}-sensitive dye Fluo-4AM, then with Aβ1-16 (220 nM) for 30 minutes, washed three times and finally incubated with Aβ1-42 (**+**). In parallel, control cells (**-**) were preincubated with vehicle alone instead of Aβ1-16. Data are expressed as mean ± SEM (n = 71 for cells treated with Aβ1-16 and 40 for cells treated with vehicle alone). These data showed that Aβ1-16 binds to a cell component (GM1) and not to the full-length Aβ1-42 protein. By saturating the GM1 sites on the cell surface before the addition of Aβ1-42, the GBD peptide prevents the specific attachment Aβ1-42 on the cell surface and thus blocks amyloid pore formation.

<u>Table S1</u>: Cholesterol and ganglioside-binding properties of full-length amyloid proteins and isolated fragments.

**Amyloid Protein** **(human except where indicated)**	**Binding to gangliosides**[a]	**Binding to cholesterol**[b]	**Amyloid pore formation**[c]
Aβ1-42	+++	+++	**Yes**
Aβ1-42 (rat)[d,e]	+/-	+++	No
Aβ1-16	+++	-	No
Aβ22-35	-	+++	**Yes**
α-synuclein1-140	+++	+++	**Yes**
α-syn34-50	+++	+	No
α-syn67-78	-	+++	**Yes**
ΔNAC α-synuclein[e]	+++	-	No
β-synuclein1-140[e]	+++	-	No

[a]Quantified by surface pressure measurements of a ganglioside monolayer at the air-water interface after the addition of the protein in the aqueous phase [1].

[b]Quantified by surface pressure measurements of a cholesterol monolayer at the air-water interface after the addition of the protein in the aqueous phase [2].

[c]Determined in this work by single cell fluorescence analysis of SH-SY5Y cells pre-loaded with the Ca^{2+}-sensitive probe Fluo-4AM and then incubated with the indicated protein.

[d]Studied in [3].

[e]Deduced from sequence data.

<u>Fig. S7</u>: Chimeric α-syn/Aβ ganglioside-binding peptide and bexarotene.

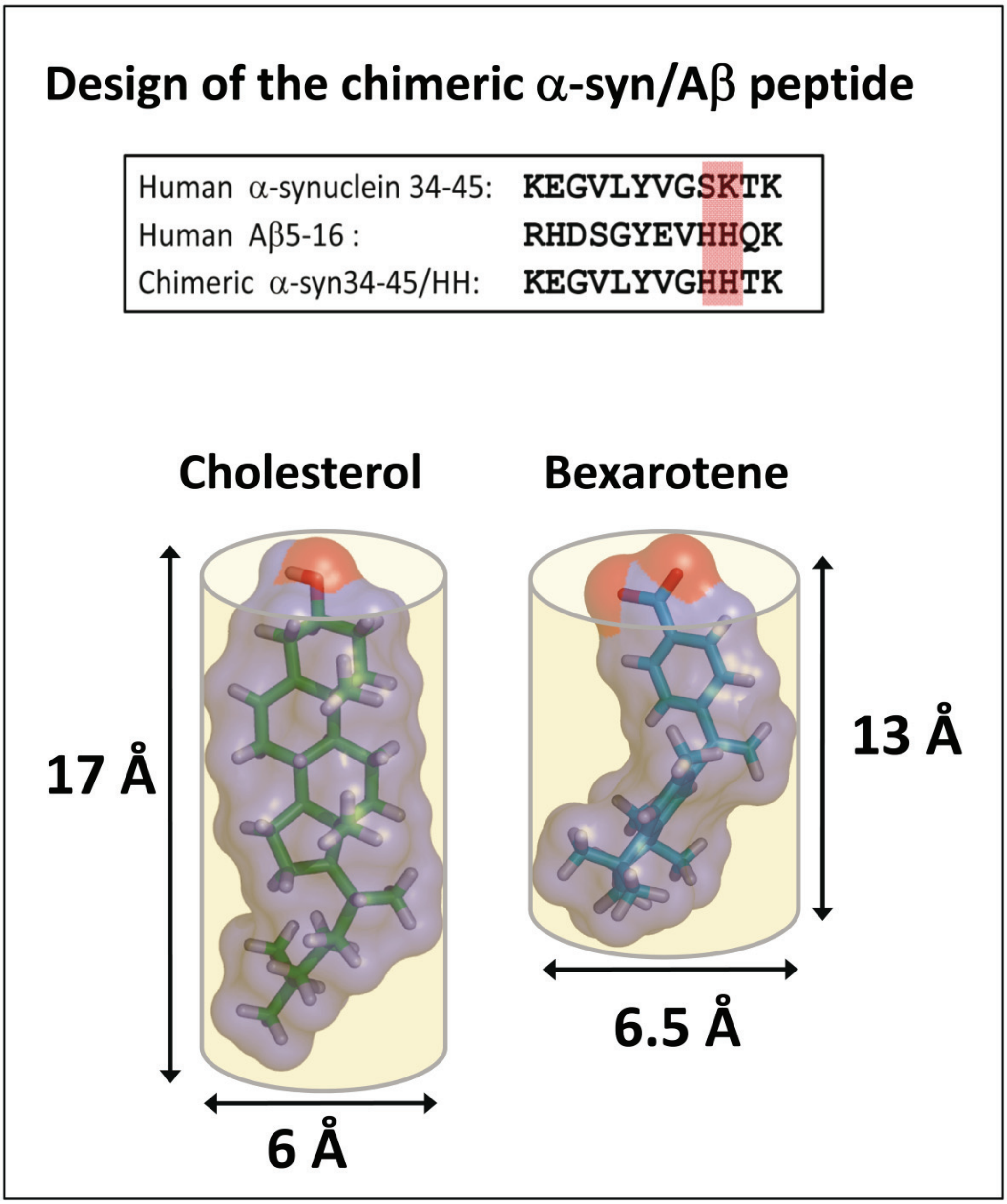

The chimeric α-syn/Aβ peptide has been described previously[1]. It recognizes all human brain gangliosides including GM1, GM3, GM4, GD1a, GD1b, and GT1b and thus blocks the initial interaction of amyloid proteins with cell surface gangliosides. The activity of bexarotene against amyloid pores has been described[4]. Both cholesterol and bexarotene share a common amphipathic structure with a large polycyclic apolar domain and a small polar head.

***Fig. S8*: Bexarotene prevents the formation of amyloid pores through competitive inhibition of cholesterol binding.**

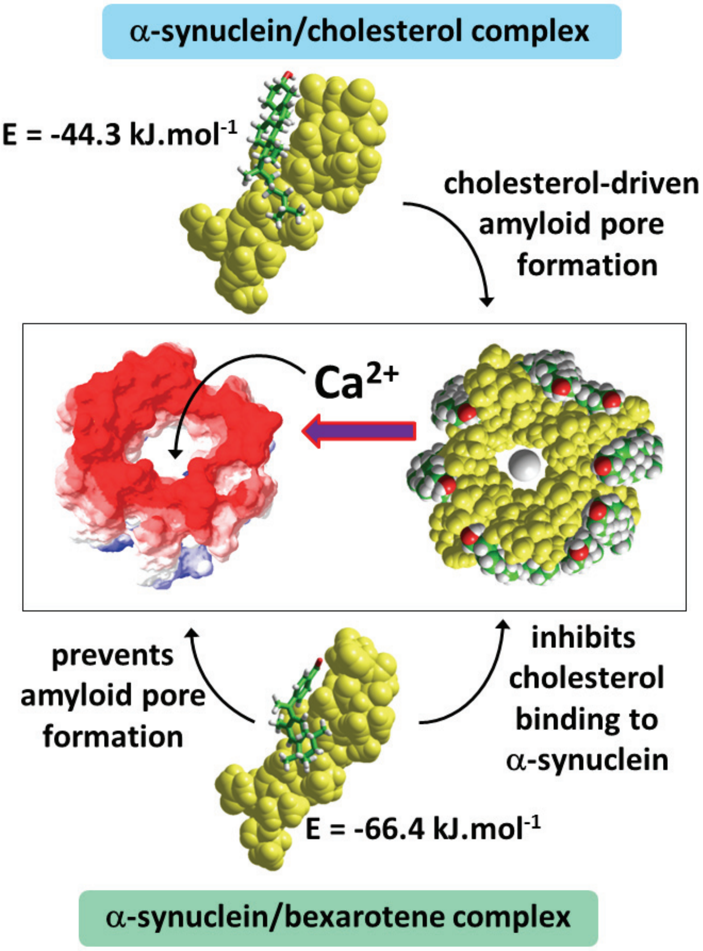

The framed middle panel represents an amyloid pore formed by the oligomerization of α-synuclein/cholesterol units[5]. It results from the binding of a cholesterol molecule on each monomer of α-synuclein (upper panel). Once formed, the pore allows massive Ca^{2+} entry into the cells. Bexarotene (lower panel) interacts with the cholesterol-binding domain of both Aβ1-42[4] and α-synuclein (region 67-79 as described in the figure). Bexarotene has a higher affinity for amyloid proteins than cholesterol, which explains why it prevents amyloid pore formation when present in competition at equimolar concentrations[5].

E, energy of interaction of the cholesterol/α-synuclein and bexarotene/α-synuclein complexes.

References.

1 Yahi, N. & Fantini, J. Deciphering the glycolipid code of Alzheimer's and Parkinson's amyloid proteins allowed the creation of a universal ganglioside-binding peptide. *PloS one* **9**, e104751 (2014).

2 Di Scala, C., Chahinian, H., Yahi, N., Garmy, N. & Fantini, J. Interaction of Alzheimer's beta-amyloid peptides with cholesterol: Mechanistic insights into amyloid pore formation. *Biochemistry* **53**, 4489-4502 (2014).

3 Ueno, H. *et al.* Comparison between the aggregation of human and rodent amyloid beta-proteins in GM1 ganglioside clusters. *Biochemistry* **53**, 7523-7530 (2014).

4 Fantini, J. *et al.* Bexarotene blocks calcium-permeable ion channels formed by neurotoxic Alzheimer's β-amyloid peptides. *ACS Chem. Neurosci.*, 5:216-224 (2014).

5 Fantini, J. & Yahi, N. *Brain lipids in synaptic function and neurological disease. Clues to innovative therapeutic strategies for brain disorders.*, Elsevier Academic Press (2015).
